# Determining Whether Agonist Density or Agonist Number Is More Important for Immune Activation via Micoparticle Based Assay

**DOI:** 10.3389/fimmu.2020.00642

**Published:** 2020-04-09

**Authors:** Peter Deak, Flora Kimani, Brittney Cassaidy, Aaron Esser-Kahn

**Affiliations:** Pritzker School of Molecular Engineering, The University of Chicago, Chicago, IL, United States

**Keywords:** toll-like-receptor, innate immunity, microparticle, activation threshold, *In vitro* quantification

## Abstract

It is unknown if surface bound toll-like-receptor (TLR) agonists activate cells via density or total molecular number. To answer this question, we developed a TLR agonist surface conjugated polystyrene microparticle (MP) system. Using a library of MPs with varying TLR agonist density and number, we simultaneously observed innate immune cell MP uptake and TNFα expression using ImageStream flow cytometry on a cell by cell basis. The data shows that total TLR number and not density drives cellular activation with a threshold of approximately 10^5^–10^6^ TLR agonists. We believe that this information will be crucial for the design of particulate vaccine formulations.

## Introduction

Toll-like-receptors (TLRs) initiate responses in the innate immune system by recognizing molecules present on the surfaces of bacteria and fungi. Given their importance for initiating an immune response, TLR agonists are widely studied for understanding innate immune responses and for their usefulness as vaccine adjuvants (1). Recently, researchers showed that TLR agonist activation can be enhanced by presenting the agonists in a particulate (2–4). TLR agonists have been packaged inside microparticles (MPs), conjugated to polymers and conjugated on MP surfaces to form “pathogen mimetic” MPs (5–10). Improvements in vaccine activity occur when agonists are attached to a particle, likely due to increases in valency and antigen proximity, but the different systems lack a basis for comparison as the particle structure, size and agonist density have not been consistent. One great example of probing agonist density and identity comes from the Roy lab, in part, but this study focused on the exciting differences *in vivo* responses (11). Therefore, to date, there has not been a direct characterization of how surface bound TLR agonists effect innate immune cell activation. Answering this question would help guide the design of future vaccines and immune-therapies that rely on particulate presentation to enhance immune responses.

**FIGURE 1:**
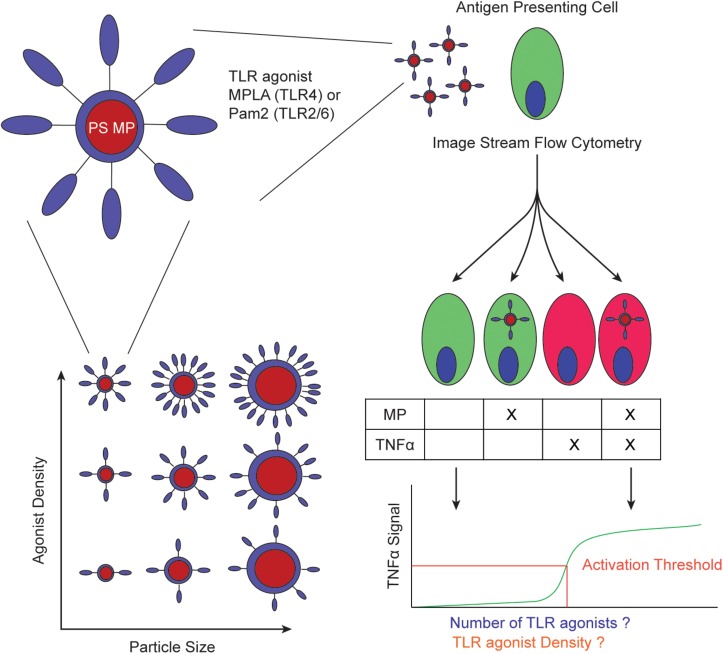
Schematic of experimental workflow. PS MPs are conjugated with TLR agonists with varying number of molecules and/or density and quantified **(left)**. These MPs are incubated with APCs to determine if the activation threshold is determined by total number of TLR agonists or TLR agonist density **(right)**.

This study seeks to quantitatively determine an activation threshold for micro-particle surface bound TLR agonists using mouse derived dendritic cells (BMDCs). A threshold for particle-surface TLR agonists could be the result of either a fixed surface density or an absolute concentration of agonists. We developed a system to answer two questions: (1) Is innate immune cell activation by surface bound TLR agonists a function of TLR agonist density on the particle or dictated by the total number of TLR-TLR agonist interactions? and (2) if one mechanism is dominant, can we quantify the threshold at which a cell becomes activated? To answer these questions, we synthesized particles of different sizes and densities and directly measured if density or absolute concentration trigger similar of different TNFα responses (Figure 1). By quantifying the number of agonists and/or the density of agonists on each MP, we correlate agonist number or density to immune activation.

## Materials and Methods

A full description of the Materials and Methods used in this study are provided in the Supplementary Material.

## Results

### TLR Agonist Conjugated Microparticle Synthesis

To achieve fine control of agonist density and absolute concentration, we developed a synthesis strategy for conjugating a TLR agonist oligonucleotide (CpG) to siloxane coated polystyrene (PS) microparticles (MPs) (12). Using a similar strategy, we conjugated PamCSK_4_ (Pam), a TLR2/6 agonist, and monophosphoryl lipid A (MPLA), a TLR4 agonist, to siloxane coated PS MPs (Supplementary Figure S1). In this study, we quantified the number of TLR agonists conjugated to MPs of varying sizes, incubated them with BMDCs and other immune cell lines and observed immune activation via TNFα secretion – a common indicator of immune action (Figure 1) (13).

### TLR Agonist Surface Conjugation Estimation

An estimate of a surface bound TLR agonist threshold has been difficult to obtain. Inconsistent conjugation and the heterogeneous nature of TLR make direct quantification of each difficult. In addition, TLR agonists activate their receptors with as low as picomolar concentrations. Some of the most potent agonists, such as lipopolysaccharide (LPS), are a heterogeneous mixture of large macromolecules of various sizes (14, 15). Additionally, given that most TLR agonists are large hydrophobic molecules, they can be challenging to chemically modify and to determine if they covalently attach to a MP or simply non-specifically associate to their surfaces (16). Finally, many polymeric MPs activate innate immune cells non-specifically and this background noise complicates analysis (12).

**FIGURE 2:**
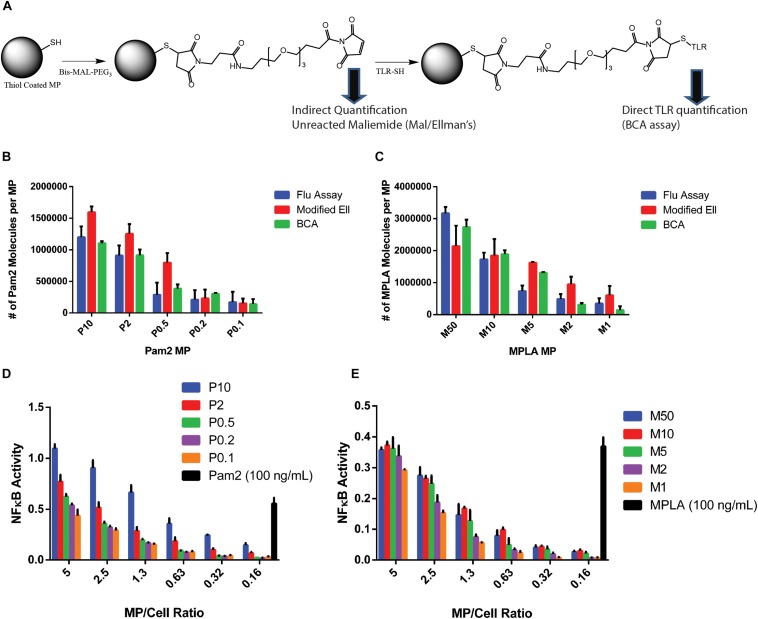
TLR agonists surface conjugation and quantification. **(A)** Chemistry schematic for MP modification with TLR ligands. **(B)** After conjugating Pam2 to 2 μm diameter MPs and washing to remove unreacted TLR agonists, the total TLR agonist conjugated to MPs were tested with the three analytical quantification methods, Fluorescence, Ellmans or BCA. **(C)** The analysis from part B was repeated with MPLA conjugated MPs. **(D)** one million RAW Blue cells were incubated with varying numbers of Pam2 conjugated MPs formulations or free Pam2 (100 ng/mL) for 16 h, then tested using a Quanti-blue SEAP reporter assay for NFkB activity. Experiments were performed as technical triplicates. The red line indicates baseline activation. **(E)** Repeated analysis of part **(D)** with MPLA conjugated MPs and 100 ng/mL free MPLA. Error bars represent standard deviation of triplicate experiments. MP labeling scheme: P for Pam2 and M for MPLA, number represents concentration of agonist during reaction in μg/mL (e.g., P10- MPs conjugated with 10 μg/ml Pam2).

To address the issues of quantification, consistency in conjugation and characterization, we employed multiple strategies. For background activation, we used polystyrene (PS) MPs with a siloxane coating – reducing the immune activation to background levels (12). We modified this PS MP system to generate MPs of various sizes, 0.25, 2, and 5 μm diameter which when coated with similar agonist concentrations yield different densities and confirmed that they do no aggregate in aqueous conditions (Supplementary Figure S2A). We carefully selected our TLR agonist system to ensure consistency and ease of characterization. MPLA and Pam2 are small (MW > 2000 Da) compared to other agonists and have well defined structures. Both MPLA and Pam2 are readily synthesized or modified for conjugation to PS MPs using maleimide-thiol chemistry (Figure 2A and Supplementary Figure S1). This chemistry is very efficient and highly consistent, specifically for surface conjugations to larger particles (16). Additionally, both molecules can be quantified via their amide bonds using a bicinchoninic acid assay (BCA).

These functionalities make the PS MP system ideal for quantitatively measuring TLR agonists conjugated on MP surfaces. This chemistry is important as accurately determining the number of agonist molecules on a MP surface is crucial to answer our overall question if TLR agonist density or number is more critical. To obtain greater accuracy, we used three independent quantification methods to count the number of agonist conjugations on the particle surface. First, we used a BCA assay to directly quantify the number of agonist molecules on the surface (17). The BCA does not interfere with particles as it relies on soluble material to quantify amide bonds. However, because the BCA measures any amide bond, the assay can result in high background measurements when testing MPs, due to non-specific absorption of molecules on the particle surface. In our case, this background was exacerbated by the presence of an amide bond in the maleimide linker. For all experiments, we subtracted a maleimide-linker conjugated MP as our control (Figures 2B,C).

To ensure accuracy, we used two other methods that measure the total number of reactive maleimides on the particle surface and determined the number of molecules on the surface via subtractive measurement. Both methods are indirect, but when combined provide further accuracy in our measurement. The first method measures the total number of free thiols on an unmodified MP and the number of maleimide molecules after reaction with the maleimide linker by observing a decrease in a known amount of L-cysteine in solution (Supplementary Figure S3). The data indicated that the maleimide conjugation is nearly 100%. To quantify the number of unreacted maleimides, we used a modified Ellman’s assay to determine a drop in a known L-cysteine concentration (16). As a third confirmation, we used a commercially available fluorescent maleimide quantification kit for both Pam2 and MPLA conjugated MPs (Figures 2B,C). We conjugated the MPs with varying concentrations of MPLA or Pam2 (50–0.1 μg/mL), which provided a large range of total agonists (3 × 10^6^–1 × 10^5^ molecules/MP).

### TLR Agonist Modified MPs Activate Immune Cells

After quantifying the number of MPLA and Pam2 molecules on the MP surface, we tested these MPs using a RAW blue activation assay to confirm that they stimulate immune cells (Figures 2D,E) (12). The RAW blue data not only shows that the MP are immunostimulatory, but also indicates a cell-MP ratio necessary to stimulate bulk immune activation. When sufficient quantities of innate immune cells are activated simultaneously, paracrine signaling often results in high levels of bulk activation, even in cells that do not encounter the activating agent (in this case the TLR agonist) (18). In our experiments, we sought to observe on a cell by cell basis if a phagocytosis event of a MP triggers activation of a cell and remove the secondary activation mechanisms of paracrine signaling. Another possibility we wished to avoid is that MPs bind their TLRs transiently and then dissociate before being phagocytosed. This transient activation is possible given that TLR2/6 and TLR4 are both surface expressed. Based on the RAW blue data, we expect that cells dosed with a 1 to 5 ratio of MPs to cells would have little to no paracrine activation and transient TLR binding, due to the shortage of MPs. Additionally, cells were dosed with a paracrine signaling blocking agent, brefeldin A (BFA), prior to MP stimulation to further reduce the non-TLR mediated cellular activation and to sequester TNFα (19).

### ImageStream Analysis of Endocytosed MPs

**FIGURE 3:**
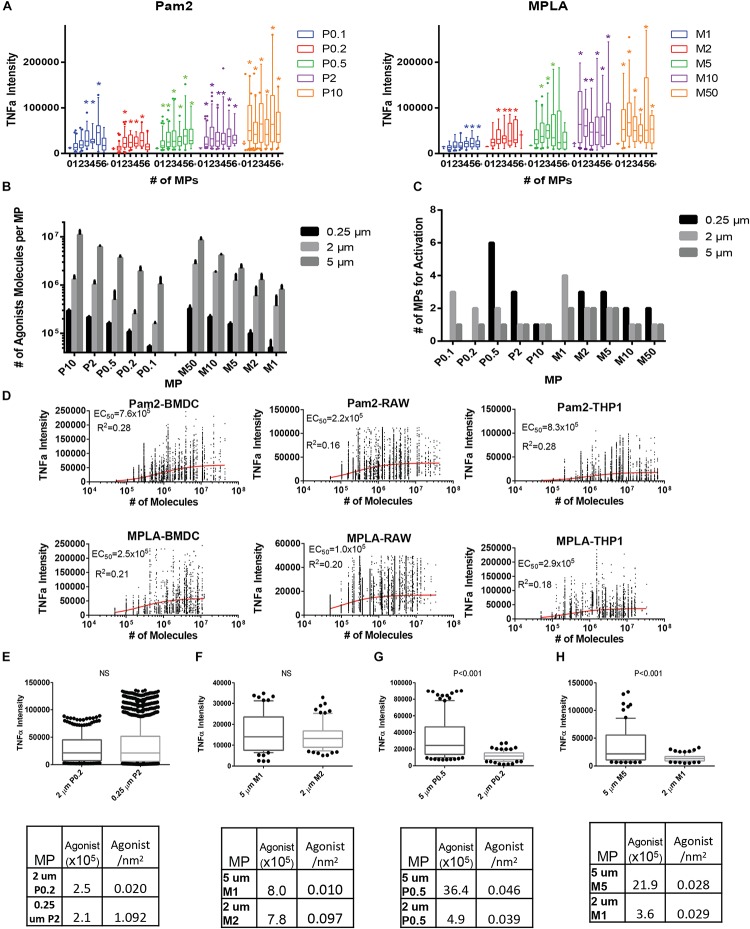
TLR coated MPs induce innate immune cell TNFα expression, which correlates with total number of TLR agonists exposed to cells. **(A)** 10 million BMDCs were incubated with two million varying Pam2 or MPLA conjugated 2 μm MPs overnight in 1 μg/mL brefeldin **(A)** BMDCs were then washed, fixed, permabilized, stained and analyzed with imagestream (>100,000 cells per run, done in triplicate then combined). TNFα expression was then compared to the number of particles which cells uptake (1, 2, 3, 4, 5, or >5 which we call 6+) and compared to baseline TNFα (the average TNFα expression of unstimulated BMDCs). Conditions with a significant (*p* < 0.05) increase in TNFα when compared to unstimulated cells are marked with a colored star. Data for Pam2 is on right and MPLA is on the left. For each condition, box and whisker plots represent one standard deviation (box) and error bars for **(A)** and **(B)** represent 90/10% range with dots >90 or <10% range, with *N* > 10 and significance as *p* < 0.05. **(B)** After conjugating Pam2 or MPLA to 0.25 and 5 μm diameter MPs and washing to remove unreacted TLR agonists, the total TLR agonist conjugated to MPs were tested with the three analytical quantification methods, Fluorescence, Ellmans or BCA, then averaged. Error bars represent standard deviation from all three testing methods. **(C)** Bar graph representing number of MPs that BMDCs uptake for each MP formulation that had a significant average TNFα signal above the average unstimulated BMDC. **(D)** TNFα expression is well correlated with total number of TLR agonists on MP surfaces. By further analyzing the imagestream data, the TNFα expression for BMDCs that uptake any number of MPs was compared with the total number of TLR agonists that interact on a BMDC surface (calculated by multiplying the number of molecules per MP by the number of MPs that cells uptake). Included are curvefits for Pam2 (top) or MPLA (bottom) MPs with BMDCs (left), RAWs (middle) or THP-1 (right). EC_50_ and *R*^2^ values were determined using a Hill curve fit model in Graphpad 7 software. **(E)** We selected two MP formulated that either had similar total number of Pam2 agonists per MP (see table below) and compared the levels of TNFα secretion when BMDCs with a single MP. Statistical difference between the two group is shown above graph, if *p* > 0.05 then it is not significant (NS). **(F)** similar to part **(E)** but for MPLA MPs **(G)** Similar analysis as in part **(E)**, but comparing two MP formulations with similar Pam2 agonist density. **(H)** Similar to **(G)** but with MPLA MPs.

To observe the number of endocytosed microparticles, we developed an ImageStream workflow to count particles within individual cells. The method also allows us to correlate TNFα production within each individual cell with the number of particles with that same cell. After a 16 h incubation, innate immune cells (BMDCs, RAW 264.7 or THP-1) were washed, fixed, permeabilized and stained for TNFα. The single cell suspension was then analyzed with Image Stream flow cytometry (Supplementary Figure S4) (20). Image Stream provides images of individual cells. With these images, we then count the number of fluorescently labeled MPs in each cell and the TNFα intensity (21). Due to the cell to MP ratio of 5:1, there was a bimodal distribution of cells, a small population of cells (<10%) that contained a MP, which was the focus of this study. To prevent skewing data toward the non-MP cells, we first confirmed that this population had a normal distribution of TNFα intensity and used only the averages for each independent experiment in all plots and significance tests (Supplementary Figure S5). In order to obtain a sufficiently large population of BMDCs with MPs, we analyzed at least 100,000 cells in biological triplicates for all variation of TLR coated MPs (Figures 3A,B and Supplementary Figure S6). BMDCs that uptake MPs with more agonist molecules per MP require fewer MPs to increase TNFα signal for both Pam2 and MPLA. This result occurred in all our test innate immune cell lines, THP-1 and RAW 264.7 (Supplementary Figures S7, S8). To readout immune activation further upstream of TNFα in cells, we used the nuclear co-localization of NFκB taken at 15 min post stimulation rather than overnight with TNFα. However, NFκB was more readily activated at even the lowest number of agonists on a single MP for both Pam2 and MPLA. This activation level suggests that NFκB nuclear colocalization requires less stimulus than TNFα secretion. For example, for the MP with the fewest Pam2 or MPLA agonists (P0.1 or M1) cells required the uptake of at least 3 MPs to trigger TNFα production, while cells only required one MP to stimulate NFκB (Supplementary Figure S9).

### Total Agonist Number on MPs More Critical for Immune Activation Than Agonist Density

To probe ligand concentration versus ligand density, we generated MPLA and Pam2 conjugated MPs of different sizes (5 μm and 0.25 μm diameter) and quantified them in a similar fashion. We estimated the ligand density for each particle by calculating the surface area of the particle (assuming a sphere, πd^2^) and dividing the total number of agonists by the surface area. We achieved a wide range of both total agonist ligands per particle and agonist density. For agonists per particle, we synthesized a range of 5 × 10^4^ to 1 × 10^7^ (Figure 3B, Supplementary Figure S10, and Supplementary Table S1). For ligand density, this data can be represented as a range of 1 × 10^4^ to 1.6 × 10^6^ molecules per μm^2^. For MP endocytosis, a similar trend was observed where more ligands per particle required fewer endocytosed MPs to trigger TNFα, but also the larger 5 μm particles with more ligands (10^6^–10^7^ molecules/MP or 10^4^–10^5^ molecules/μm^2^) required only either 1 or 2 MPs for each condition and smaller 0.25 μm particles (5 × 10^4^–3 × 10^5^ molecules/MP or 2.5 × 10^5^−1.5 × 10^6^ molecules/μm^2^) required over 1 MP for all but one condition, P10 (3 × 10^5^ molecules/MP or 1.5 × 10^6^ molecules/μm^2^, Figure 3C and Supplementary Figures S11–S13).

With the insight that density and ligand number were both variables, we analyzed a complete data set to observe larger trends. We compared the correlation between agonist density on MPs or total number of agonist-TLR interaction and TNFα intensity. To estimate the total number of TLR agonists encountered by a BMDC, we estimated each particle could fully expose its agonists to the endocytic environment. We calculated the agonists per MP by multiplying the total number of MPs phagocytosed by a BMDC for all MP sizes and agonist conjugation ratios. We then plotted this data with the corresponding TNFα intensity and performed a curve fit analysis using Graphpad Prism 6 (Figure 3D). Both MPLA and Pam2 have a highly significant positive trend between number of agonists and TNFα intensity (*p* < 10^–3^) and reasonable *R*^2^ values for the curve fit given the degree of biological variability inherent in an immune response (Table 1). Moreover, we observed no significant trend when the TNFα intensity was compared to agonist density on MPs (Supplementary Figure S14), indicating that BMDC activation is largely a function of total agonist molecules and not agonist density on MP surfaces. This trend is also seen in other cell lines, such as THP-1 and RAW 264.7 cells (Figure 3D and Table 1).

**TABLE 1:** List of TNFα activation threshold.

**Cell Line**	**BMDC**			**RAW**			**THP-1**		
**Agonist**	**EC_50_ (×10^5^) molecule**	**CI (×10^5^) molecule**	***R*^2^**	**EC_50_ (×10^5^) molecule**	**CI (×10^5^) molecule**	***R*^2^**	**EC_50_ (×10^5^) molecule**	**CI (×10^5^) molecule**	***R*^2^**
Pam_2_	7.6 ± 0.5	6.6−8.6	0.28	2.2 ± 0.1	2.0−2.5	0.16	8.3 ± 0.5	7.3−9.3	0.28
MPLA	2.5 ± 0.2	2.1−2.9	0.21	1.0 ± 0.06	0.9−1.1	0.20	2.9 ± 0.17	2.5−3.2	0.18

We next sought to determine if density was impacting cell activation in any quantifiable way. While the correlation between the numbers of MP bound TLR agonists and immune activation is clear in the large analysis, the high biological variability of innate activation partially masked a more interesting phenomenon when comparing individual sets. In essence, the variability in cell activation across sets meant that statistical analysis had much greater power when comparing individual sets. To determine if density had an effect, we directly compared systems with different agonist densities but similar total number of agonists. As shown in Figure 3E, the 2 μm P0.2 MP (2.4 × 10^6^ molecules/MP, 2.0 × 10^4^ molecules/μm^2^) has a similar total number of agonists per MP as the 0.25 μm P2 MP (2.1 × 10^6^ molecules/MP, 1.1 × 10^6^ molecules/μm^2^). BMDCs that endocytose a single MP in each case have a similar levels of TNFα activation. This effect is also seen in MPLA MPs (Figure 3F). In contrast, MPs with similar levels of agonist density have dissimilar TNFα intensity (Figures 3G,H). For example, the 5 μm M5 MP (2.1 × 10^6^ molecules/MP, 7.0 × 10^3^ molecules/μm^2^) and the 2 μm M2 MP (3.6 × 10^5^ molecules/MP, 7.2 × 10^3^ molecules/μm^2^) had similar agonists densities and dissimilar total agonist numbers but had a statistically significant difference in TNFα expression (Figure 3H, *p* < 0.001). We observed these relationships in all MP density comparisons (Supplementary Figure S15) – comparing across particle size and total agonist numbers. Based on this data, we concluded that the immune activating potential for TLR agonists when bound to a MP is correlated more with the total number of agonists on the particle and not with the density of agonist conjugation.

In an effort to provide further insight, we estimated the order of magnitude for the activation threshold (the number of agonists required to engage TLRs) to trigger innate immune activation. We calculated an EC_50_ value to estimate the activation threshold for BMDCs by plotting the number of molecules per particle compared to the TNFα intensity (Figure 3D). This estimate varies slightly between the TLR agonist used (7.6 × 10^5^ for Pam2 vs. 2.5 × 10^5^ for MPLA). This estimate likewise varies slightly for THP-1 or RAW 264.7 cells (Table 1). Despite these small variations, we generate a range between 10^5^–10^6^ MPLA or Pam2 molecules required on MP surfaces to activate an innate immune cell. This information provides a minimum guideline for the design of any surface bound TLR agonist vaccine formulation and suggests a range of activation for APCs more generally.

## Discussion

This result strongly suggests that the total number of ligand-receptor interactions is the primary driver of antigen presenting cell activation and not the density of ligands. While this evidence strongly supports agonist number, it does not rule out that particle density can influence immune responses in other ways. TLR agonist density alters downstream responses including Th1/Th2 bias, CD8 T-cell responses, and Fc receptor density (10, 22–24). Taking into account these finding, we propose a potential two-step process in which a cell is activated and then uses density and concentration to inform the type of response that develops.

It should be noted that this type of analysis has some limitations. Namely, our estimate is not a description of the total number of agonist-TLR interactions required to initiate downstream immune activation, rather it is an empirical observation of the number of agonists required on the MP surface to stimulate innate immune cells. Determining a quantitative estimate of the number of agonist-TLR interactions is challenging because the total number of TLR on APCs has not been definitely determined and obtaining estimates of total number of binding events in such highly multivalent systems as MPs is notoriously difficult (25). Instead, our analysis sidesteps these issues and focuses exclusively on quantifying the number of TLR agonists necessary to generate APC activation. While this analysis lacks mechanistic insights on TLR mediated APC activation, it provides more of a practical guideline for particulate vaccine formulations.

For this study, we provide the following overall insights into particulate immune activation, namely (1) initial TLR mediated APC activation has a threshold dictated by the absolute number of TLR agonists and not density and (2) to activate an APC, a particle must have on the order of 10^5^–10^6^ TLR agonists on its surface. To test this, we employed a PS MP based system, which allowed for discrete and quantifiable conjugation of molecules per MP and a mechanism for tracking which cells bind TLR agonists (via fluorescence). In combination with ImageStream flow cytometry, this MP system reliably tracked innate immune cell activation and determined a TLR agonist activation threshold. The analysis from this approach is limited to observing just APC activation, but future work will track downstream adaptive immune responses. Using the MP system, we envision stimulating APCs with MPs for T-cell expansion and *in vivo* experiments. TLR agonist MP formulations are a promising new direction for vaccine development and have many potential applications to enhance current vaccine formulations (11). These types of quantitative measurements will aid the design of these MPs, allowing optimal immune activation while reducing extraneous TLR agonists and excess inflammation which contribute to reduced tolerability in vaccines and potential side effects in immune-therapies.

## Data Availability Statement

All datasets generated for this study are included in the article/Supplementary Material.

## Ethics Statement

The animal study was reviewed and approved by the University of Chicago IACUC.

## Author Contributions

PD and AE-K designed the study and developed the initial ideal. BC performed the SEM microparticle analysis. FK synthesized, purified, and performed the analysis on thiol modified Pam_2_ molecules. PD performed all other experiments, synthesized the other compounds, performed the analysis, created the figures, and wrote the text of the manuscript. PD and AE-K edited and provided the feedback on the manuscript.

## Conflict of Interest

The authors declare that the research was conducted in the absence of any commercial or financial relationships that could be construed as a potential conflict of interest.

## Abbreviations

BCA: bicinchoninic acid assay
BMDC: mouse derived dendritic cells
CpG: oligonucleotide
MP: microparticle
MPLA: monophosphoryl lipid A
Pam_2_: Pam_2_CSK_4_
PS: polystyrene
TLR: toll-like-receptor.
