# Supplementary material for: Determining Whether Agonist Density or Agonist Number Is More Important for Immune Activation via Micoparticle Based Assay
Source: Front Immunol. 2020 Apr 9;11:642. doi: 10.3389/fimmu.2020.00642 (PMC7161694; doi:10.3389/fimmu.2020.00642)
Supplement: Supplementary file 1 [file Data_Sheet_1.PDF]

Supplementary Material

1 Supplementary Materials and Methods

Materials: All chemicals were purchased from Sigma unless noted. 0.25 and 5 μm diameter particles were purchased commercially from Sphereotech. MPLA and Pam_2 were purchased from Invivogen. BCA assay kit was purchased from Thermo and Maleimide Detection Kit (Modified Ellman's assay kit) was purchased from Sigma. Fluorescent Maleimide Detection Kit was purchased from aatBioquest (SunnyVale, CA, USA). Cytoperm/CytoFix Kit plus GolgiPlug was purchased from BD Biosciences. APC/Cy7 anti-mouse TNF-α [MP6-XT22] and Alexa Fluor® 647 anti-human TNF-α [MAb11] were purchased from BioLegend. Anti-NF-kB p65 antibody (ab16502) was purchased from Abcam. Goat-anti rabbit AF647 was purchased from Thermo. Quanti Blue assay solution was purchased from Invivogen.

Methods:

Silica-silane coated Polystyrene Microparticle (MP) synthesis: PS MPs were synthesized and coated with a silica coating using a procedure from Moser et al.[1] Briefly, uniform, spherical, 2 μm diameter polystyrene microparticles were synthesized via controlled styrene polymerization. 2 g of polyvinylpyrrolidone, MW 40,000 and styrene (20 g), washed with NaOH and dried with MgSO4, was dissolved in EtOH (250 mL) and purged with nitrogen. AIBN (0.2 g) was added, the mixture stirred at 70 °C and 200 rpm for 24 hrs. Mixture was purified by centrifugation (5000 RPM for 5 minutes, followed by washing 3X in 30 mL of EtOH to remove residual monomer, initiator, and stabilizer). 0.25 and 5 μm diameter particles were purchased commercially from Sphereotech. The surfaces of all three sizes of MP were modified with reactive thiol groups via Pickering emulsion reaction. A mixture of Cyclohexane (45 mL), n-hexanol (10.8 mL), endotoxin free water (2 mL) and Triton X-114 (10.8 mL) were placed in a round-bottom flask and sonicated for 20 min. Particles (0.2 g) were added and the suspension was sonicated for 40 min. TEOS (400 μL) was added dropwise followed by 14 M aqueous ammonia (1.2 mL). The resulting solution was stirred for 30 min RT. Subsequently, 3-Mercaptosilane (200 μL) was added dropwise and stirred for 6 h. TEOS-mercaptosilane copolymer coated particles were then pelleted at 3400 rpm for 30 min and washed 3× with EtOH. Particles were dried at 70 °C and stored at 4 °C.

Thiol modified Pam_2CSK_4 Synthesis: Synthesis was performed using a Liberty Blue™ automated peptide synthesizer. Rink amide resin (100-200 mesh, 0.55 mmole/g, 0.05 mg) was weighed out into a solid-phase peptide synthesizer reaction vessel. The peptide was constructed by coupling Fmoc-Cys((RS)-2,3-di(palmitoyloxy)-propyl)-OH, Fmoc-Ser(tBu)-OH, Fmoc-Lys-OH, Fmoc-Gly-OH, Fmoc-Cys(Trt)-OH (0.2M in DMF) from the C terminus to the N terminus. Deprotection was performed using 20% piperidine in DMF. Coupling was performed after activation with diisopropylcarbodiimide (DIC) (0.5M in DMF) in the presence of Ethyl cyanohydroxyiminoacetate (oxyma) (1M in DMF). Fmoc-Cys((RS)-2,3-di(palmitoyloxy)-propyl)-OH was coupled at 90 ^{o}C for 10 min. All other couplings were done at 90 ^{o}C for 5 min. All reactions and subsequent washes were performed in DMF. After the synthesis was completed, the resin was transferred into a Bio-Rad Poly-Prep chromatography column.

Global deprotection was achieved by agitating the resin in trifluoroacetic acid (TFA)/3,6-dioxa-1,8-octanedithiol (DODT)/triisopropylsilane (TIPS)/H2O (8.5:0.5:0.5:0.5) for 2 h. The peptide was precipitated by adding the cleavage cocktail filtrate to 30 mL diethyl ether in a 50 mL centrifuge tube pre-cooled to -78 ^{o}C. The precipitate was collected by centrifuge (4000 XG for 5 min). The precipitate was dissolved in 20% CH_3CN in 0.1%TFA) and filtered through a 0.45 µm syringe filter. Purification was performed using reversed-phase HPLC C8 column (gradient elution with 30–90% CH_3CN /0.1% TFA over 20min). Pure fractions were pooled together and the peptide was recovered through lyophilization. MALDI-TOF MS (m/z) 1431.29 [M+H] +

Thiol modified MPLA synthesis: 1 mg of MPLA and 10 mg of p-maleimidophenyl isocyanate were dissolve in anhydrous DMF (400 uL) and reacted at room temperature overnight. Then 400 uL of PBS was added to the vial with 20 uL of 2,2'-(ethylenedioxy)diethanethiol (4 mg) and allowed to react under argon for 24 hrs at room temperature. Product was lyophilized and purified with liquid-liquid extraction of DCM/water. The organic layer was dried with roto-evaporation and verified for purity with HPLC (>95%). Electro-spray mass spectroscopy confirmed the mass (m/z) 2158.97 [M+H]+

TLR agonist Surface Functionalization: Thiol bearing MPs were functionalized with MPLA and Pam_2 using thiol-maleimide chemistry. First MPs (5 mg) were swelled in ACN (500 uL) for 30 mins under sonication, then 1 mg of FITC in 500 uL of ACN was added for a final concentration of 1 mg/mL for 30 mins. MPs were then centrifuged for 1 min (5000 rcf), supernatant removed and washed 3x with PBS. FITC labeled MPs were dissolved in 500 uL of PBS, then 5 mg of Bismaleimide-PEG_3 was added and allowed to sonicate for 30 minutes, then washed 3x in PBS. After washing, maleimide bearing MPs were incubated with thiol functionalized MPLA or Pam_2 at varying concentrations in PBS (500 uL) and sonicated for 2 hrs, then washed 10 times, 3x with PBS, 4x with PBS with 0.1 tween 20, and 3x with PBS. MPs were diluted in PBS + 0.05 %wt tween20 at 20, 50 and 100X and then number of MPs in 10 uL counted using flow cytometry to determine a concentration. MP solutions were diluted to a concentration of 1 million MPs per uL of PBS and stored at 4ºC.

SEM of Functionalized Particles: Scanning electron microscopy (SEM) and energy-dispersive spectroscopy (EDS) of the particles was performed using an FEI Quanta 3D FEG dual beam (SEM/FIB) equipped with Inca EDS (Oxford Instruments). High-resolution images were taken with an FEI Magellan 400 XHR SEM particle samples were dried under vacuum for 24 h, mounted on carbon tape, and sputter coated (South Bay Technologies) with approximately 2–4 nm of Au/Pd 60:40 or Ir.

Particle Concentration Determination via Flow Cytometry- The concentration of particle in a given volume was determined using flow cytometry (NovoCyte flow cytometer, ACEA Biosciences, Inc). After the final wash, MPs were rehydrated in 500 µL of PBS, then diluted down in PBS +0.01 %wt tween 20 by 50, 100 and 200 fold. 10 µL of these dilutions were analyzed via flow cytometry in triplicate, gating on FITC signal to remove noise. The MP concentration in the stock was then calculated using linear regression from the three dilutions. MP stocks were then diluted down with PBS to a final concentration of 1 million MPs/µL.

Quantitative Determination of Agonist Molecules Per MP: The number of MPLA or Pam_2 molecules on each particle was determined using three independent assays. 1) Bicinchoninic Acid (BCA) assay; 2) Fluorescence maleimide quantification assay; 3) modified Ellman's assay. The number of

agonists per MP was calculated by taking an average of the calculated values from each of these three assays.

BCA assay- This was performed according to manufacturer's instruction (Thermo Fischer) with some modifications. 100 million beads were incubated with BCA solution and reacted for 30 mins at 60 ºC then analyzed every hour using a Multiskan FC plate reader (Thermo Scientific) and absorbance was measured at 562 nm and compared to a standard curve of modified MPLA or Pam_2 after subtracting a background of maleimide modified MP. This assay was performed with technical triplicates.

Fluorescence maleimide quantification assay- This was performed according to manufacturer's instructions (aatbio). 10 million beads were incubated in each test, removed via centrifugation and the supernatant analyzed with a Multiskan FC plate reader (Thermo Scientific). The numbers of TLR agonists per MPs were indirectly determined by comparing the drop in maleimide signal compared to a maleimide functionalized control with no TLR agonist. This assay was performed with technical triplicates.

Modified Ellman's Assay- This assay was performed using a kit according to the manufacturer's instruction (Sigma) with some modifications. After using the kit to determine the number of unreacted maleimides on the MP surface, this number was compared to a maleimide functionalized control with no TLR agonist to indirectly determine the number of TLR agonists. This assay was performed with technical triplicates.

RAW Blue NF-κB Assay: RAW-Blue NF-κB cells (Invivogen) were passaged and plated in a 96 well plate at 100k cells/well in 180 µL DMEM containing 10% HIFBS. Cells were incubated at 37 °C and 5% CO_2 for 24 h. 100 ul of cells were incubated with varying ratios of MPs at 37 °C and 5% CO2 for 18 h. After 18 h, 20 µL of the cell supernatant was placed in 180 µL freshly prepared QuantiBlue (Invivogen) solution and incubated at 37 °C/5% CO2 for up to 2 h. The plate was analyzed every hour using a Multiskan FC plate reader (Thermo Scientific) and absorbance was measured at 620 nm.

Image Stream MP Uptake Analysis for TNFα Expression: 1 million BMDCs, RAWs or THP-1s were incubated with 200,000 MPs in 1 mL of cell culture media (DMEM supplanted with 10% HIFBS) for 1 hour. After 1 hr, protein export was inhibited using a GolgiPlug Kit (BD Biosciences) with Brefeldin A according to manufacturer's instructions for 16 hrs at 37 ºC under 5% CO_2. Cells were then washed and fixed and permeablized with a BD Cytofix/Cytoperm Plus Kit (BD Biosciences). Cells were stained with a solution of anti-TNFα (1:500 dilution) and their nuclei stained using Hoechst 33342 Solution (2 µM final concentration) in permeablization buffer for 1 hr. Cells were washed 3x in PBS with 2% HIFBS, concentrated into a 20 µL volume and analyzed with ImageStream Flow Cytometry. Each MP condition was performed in triplicate, analyzing >100,000 cells per run using the gating strategy defined in Figure S-4. MPs were identified using the "Particle Count" wizard in the IDEAS software and compared to TNFα intensity per cell.

Image Stream MP Uptake Analysis for NF-κB Expression: 1 million BMDCs, RAWs or THP-1s were incubated with 200,000 MPs in 1 mL of cell culture media (DMEM supplanted with 10% HIFBS) for 15 minutes. Immediately cells were fixed using ice cold Cytofixation Buffer (BD Biosciences) for 15 minutes. Cells were then fixed in PBS +0.04% triton X for 3 minutes, immediately spun down (400 RCF, 5 minutes), supernatant removed and washed with CytoPerm Buffer (BD Biosciences) 2 times (200 µL per wash). Cells were then stained with Rabbit anti-NF-κB p65 (1:500 dilution) for 1 hr on ice, washed 3x, then stained with a secondary goat anti-rabbit AF647

(1:1000 dilution) for 1 hr on ice, washed, concentrated into a 20 μL volume of PBS with 2% HIFBS and analyzed with ImageStream Flow Cytometry. Each MP condition was performed in triplicate, analyzing >100,000 cells per run using the gating strategy defined in Figure S-4. MPs were identified using the “Particle Count” wizard in the IDEAS software and compared to NF-κB nuclear colocalization using the “Colocalization” wizard in the IDEAS software.

Data Analysis: ImageStream data was first analyzed in the IDEAS software (Amnis) for nuclear colocalization and particle counting using built-in analysis wizards. Single Cell data was then exported into Graphpad Prism 6 software for further analysis. Cell data was divided into the following categories: 0 MP, 1 MP, 2 MP, 3 MP, 4 MP or 5 MP or >5 MPs (represented as 6+ on all figure legends). Outliers were removed using the ROUT method (Q=1.0 %) for each group. For the 0 MP group, given these were the majority of cells analyzed (>90%) and they followed a normal distribution (Figure S-4), they were averaged for each experiment and plotted as three individual points so as not to skew results. For other groups, these were combined among all three triplicates to increase cell counts. If a condition had >10 cell, it was removed from analysis. Each MP bearing group was then compared to the 0 MP group for a cell line using a Mann-Whitney nonparametric test and a p value calculated for either TNFα intensity or NF-κB colocalization score. Two groups were considered significant if $p<0.05$.

2 Supplementary Figures

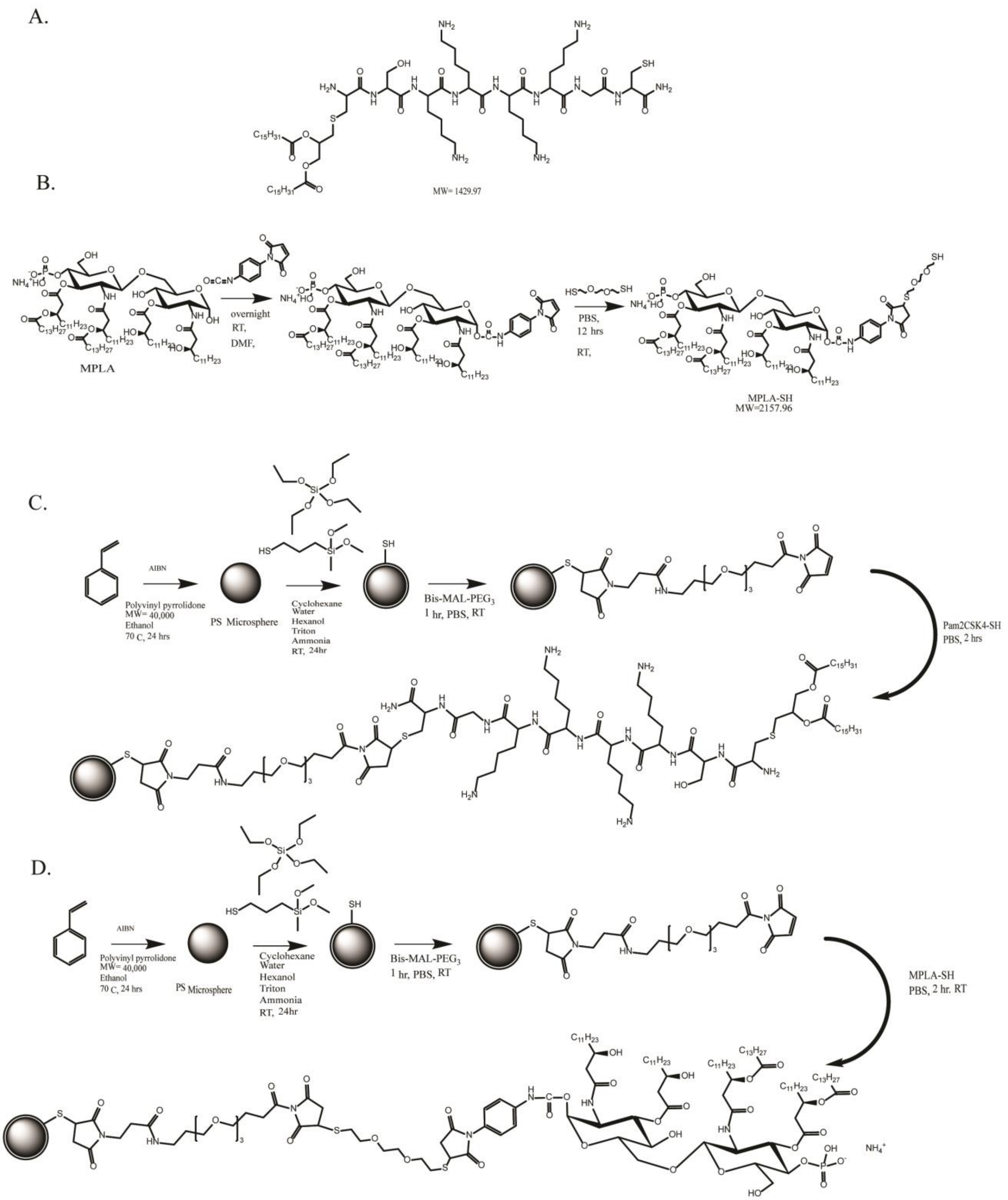

Figure S-1. Detailed schematic of chemical modification of PS MPs. (A) Chemical structure of PamCSK4- SH. (B) Reaction schematic of modified MPLA. Reaction schematic of MP conjugated with (C) Pam2CSK4 and (D) MPLA.

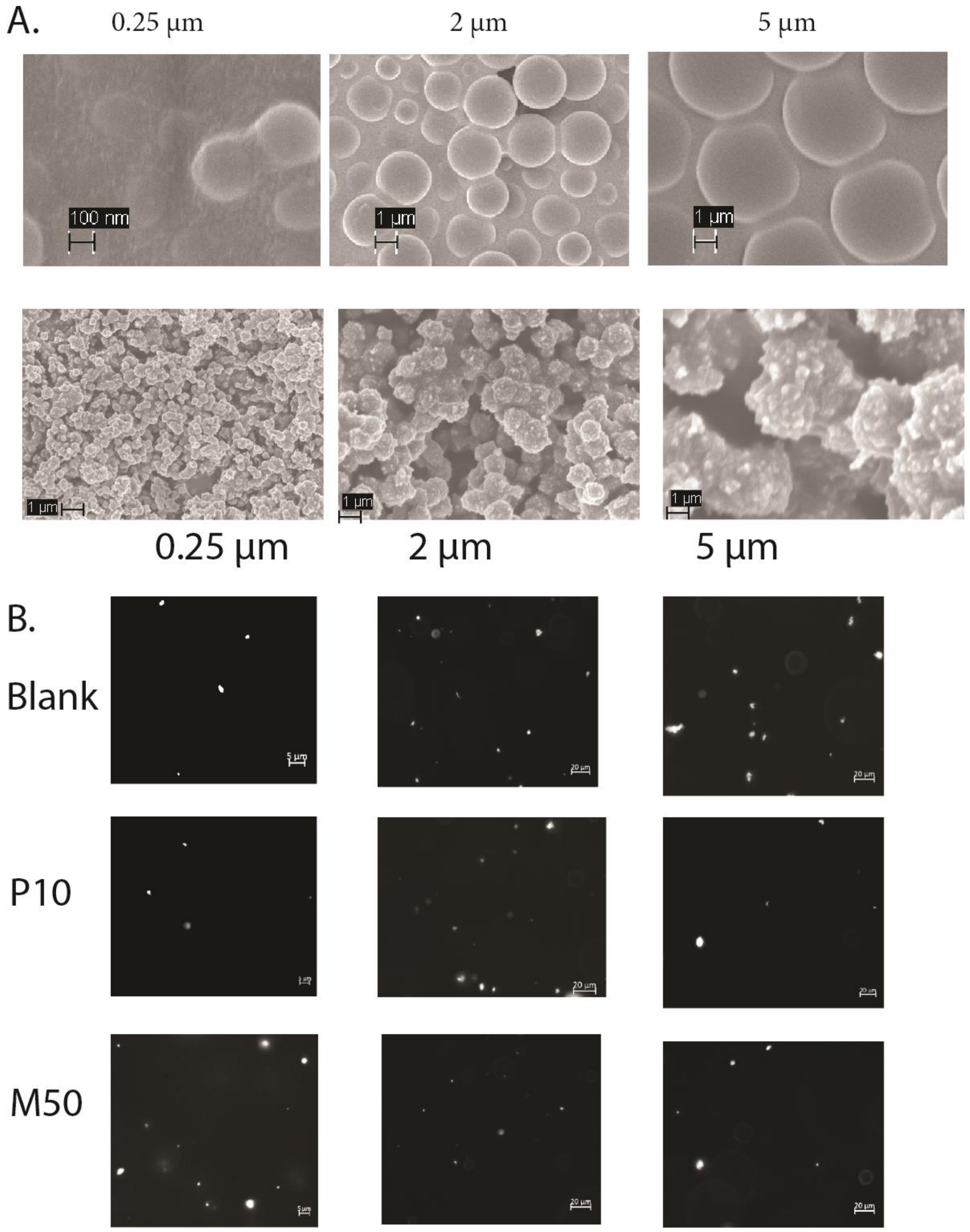

Figure S-2. (A) SEM Images of 0.25, 2 and 5 μm PS MPs. PS MPs (top) or P10 coated MPs were coated with siloxane coating, washed 5x and then mounted on SEM imaging slide. (B) MPs do not form large aggregates as shown by fluorescent microscopy. 10 uL of 1 million MPs per mL of PBS per placed on a cover slip and imaged. Blank, P10 and M50 of all three sizes of MPs were imaged to show that hydrophobic TLR agonists do not induce undue clustering of MPs in aqueous solution.

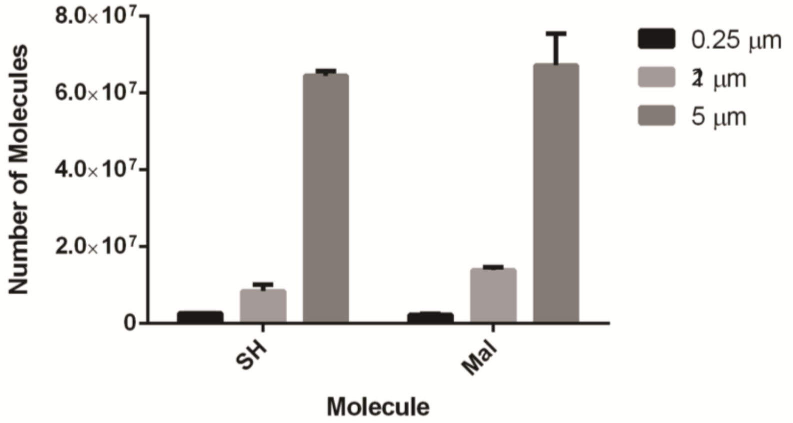

Figure S-3. Average Number of Thiol Groups per MP. 10 million thiol coated PS MPs were tested with Ellmans assay to determine total number of thiol molecules. Similarly, thiol conjugated PS MPs were incubated with bismaleimide PEG3 (1 mg per 1 mg MP) in PBS for 1 hr then tested with reverse Ellmans assay to determine active number of maleimide groups.

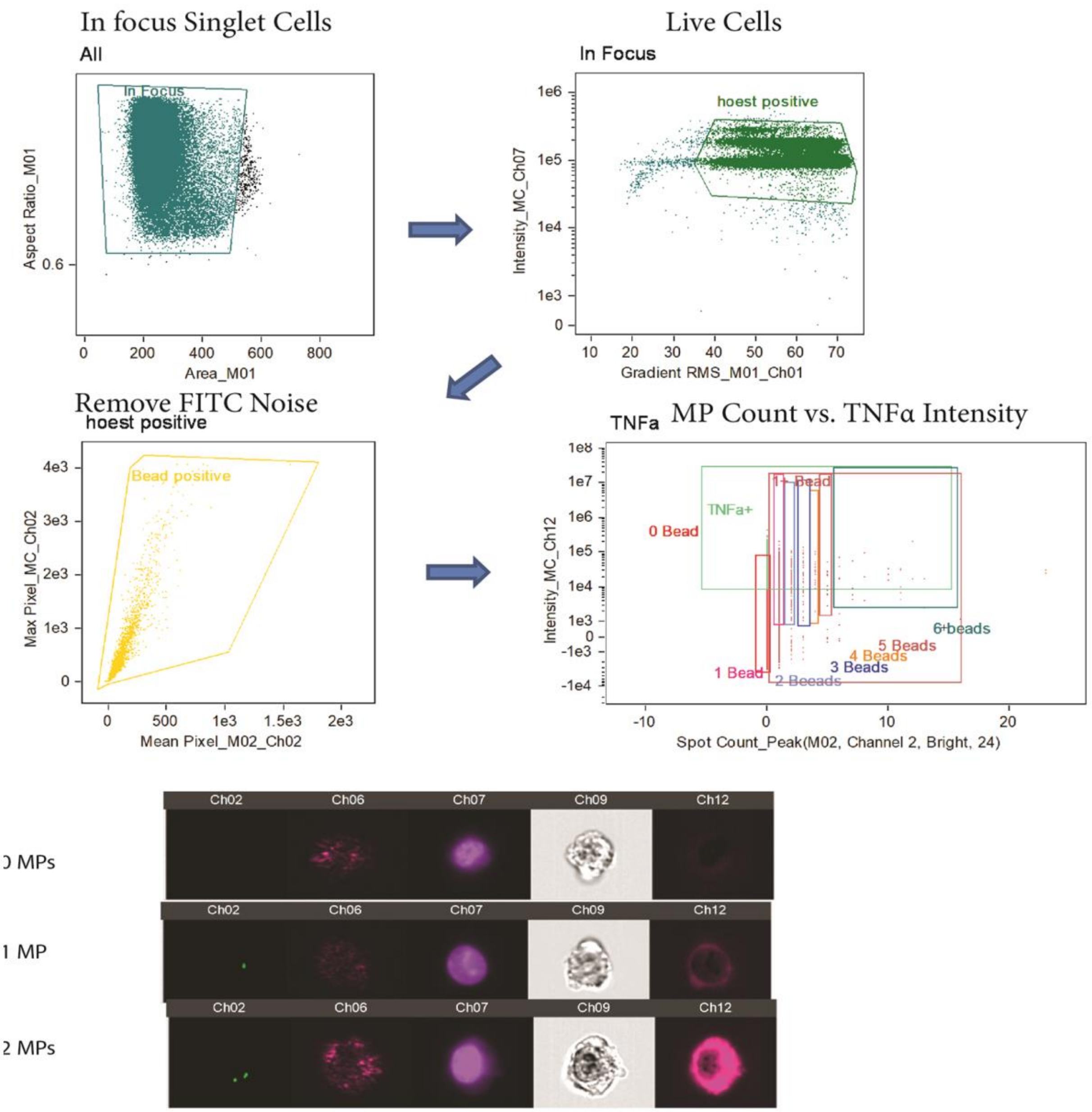

Figure S-4. ImageStream Gating Strategy. Out of focus cells and non-singlets were gated out based on size and the aspect ratio of camera 1. These cells were then gated on hoest positive and in focus for hoest for live cells. These cells were plotted for FITC intensity (Ch2) and FITC max pixel signal and the cells with high FITC signal and low FITC max signal were sorted out to remove cells with non-specific and/or background FITC signal. Finally, using the Spot Counting wizard in the IDEAS software package, the number of MPs each cell uptakes was determined and plotted against TNFα intensity (Ch12).

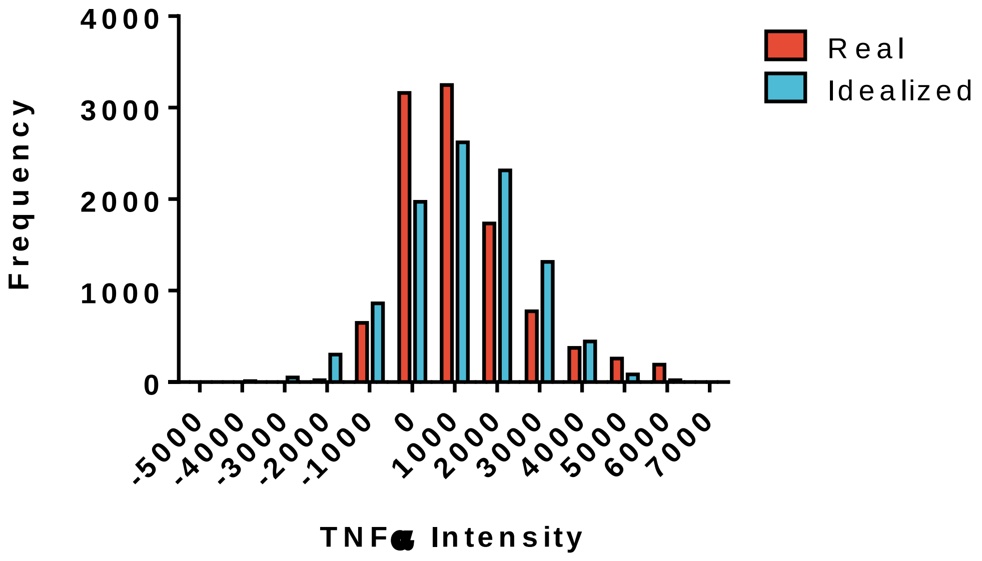

Figure S-5. Cells that do not uptake MPs have a normal distribution of TNFα intensity. BMDCs were incubated with 2 μm diameter P0.2 MPs for 16 hrs under brefeldin A treatment then analyzed for TNFα secretion via imagestream. We analyzed the cell with no MP uptake by placing them into bins for TNFα intensity and compared this data to an idealized dataset with perfect standard deviation (idealized) of the same number of total cells (10,140) generated with an online calculator with the same mean and SD and the experimental dataset (real). These two datasets were very similar ($p>0.95$, students t test) indicating that cells with no MPs have a standard deviation of TNFα intensity. This result was also seen in all other MP incubated cells datasets (data not shown).

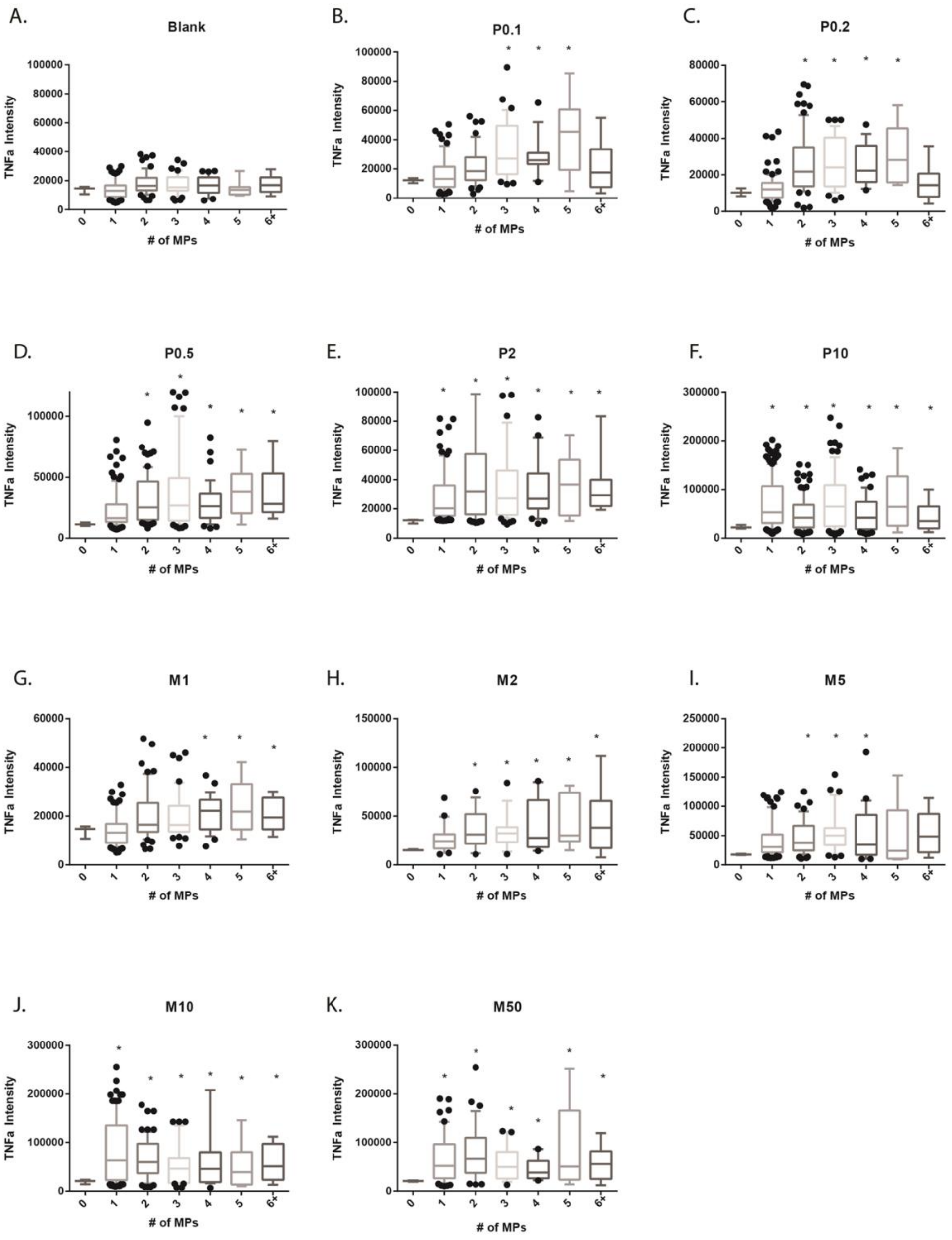

Figure S-6. BMDCs increase TNFα expression upon particle uptake. 10 million BMDCs were incubated with 2 million varying MPs overnight in 1 ug/mL brefeldin A. BMDCs were then washed, fixed, permabilized, stained and analyzed with imagestream (>100,000 cells per run, done in triplicate then combined). TNFα expression was then compared to the number of particles that cells uptake and compared to baseline TNFα (the average TNFα expression of unstimulated BMDCs). Error bars represent 90/10 % data range. Conditions with a significant ($p<0.05$) increase in TNFα

when compared to unstimulated cells are marked with a star. The MPs used were (A) Blank MPs, (B) P0.1. (C) P0.2, (D) P0.5, (E) P2, (F)P10, (G) M1, (H) M2, (I) M5, (J) M10, (K) M50.

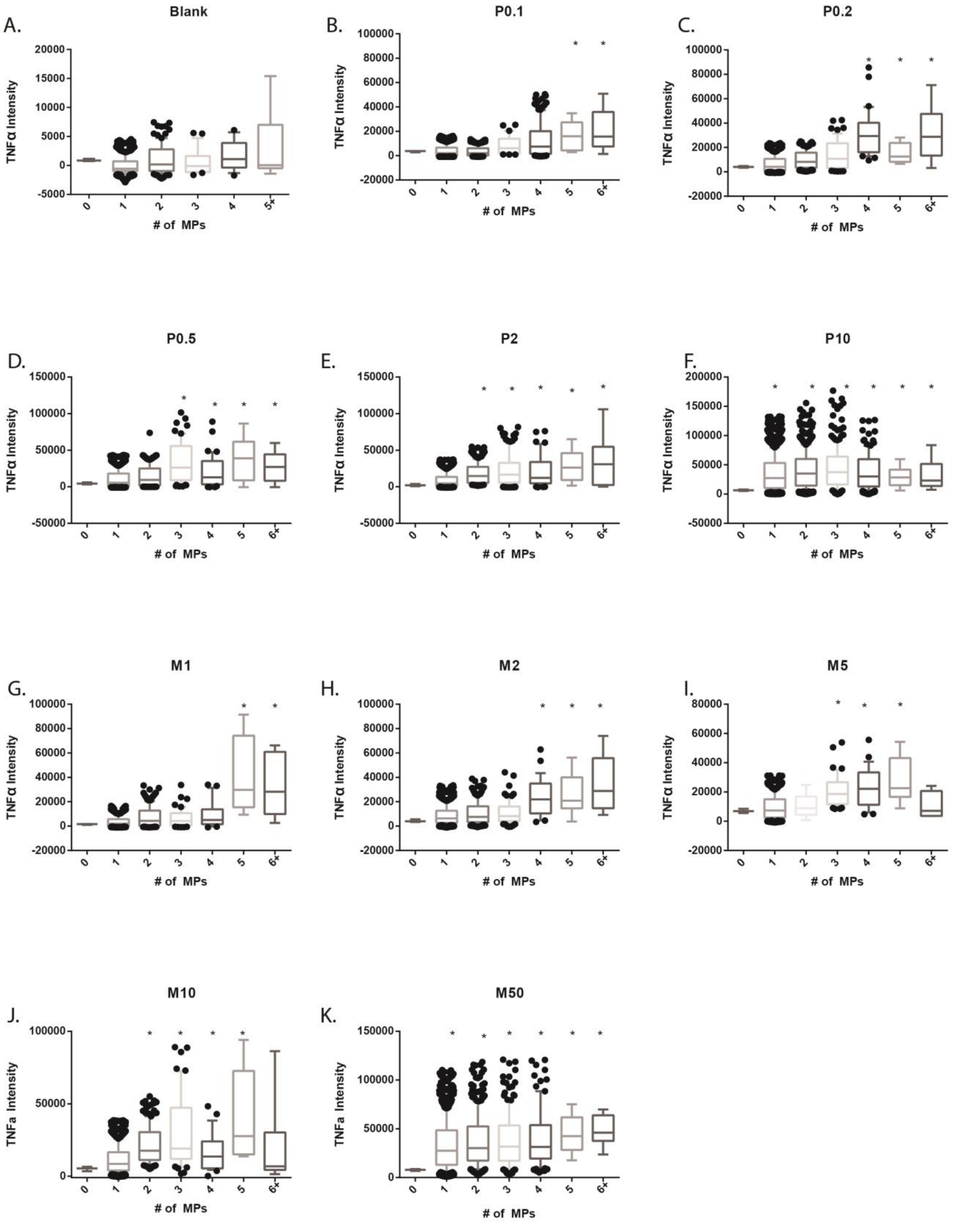

Figure S-7. RAW 264.7s increase TNFα expression upon MP^{TLR} uptake. 10 million RAWs were incubated with 2 million varying MPs overnight in 1 ug/mL brefeldin A. RAWs were then washed, fixed, permabilized, stained and analyzed with imagestream (>100,000 cells per run, done in triplicate then combined). TNFα expression was then compared to the number of particles cells uptake and compared to baseline TNFα (the average TNFα expression of unstimulated RAWs). Error bars represent 90/10 % data range. Conditions with a significant ($p<0.05$) increase in TNFα when compared to unstimulated cells are marked with a star. The MPs used were (A) Blank MPs, (B) P0.1. (C) P0.2, (D) P0.5, (E) P2, (F)P10, (G) M1, (H) M2, (I) M5, (J) M10, (K) M50

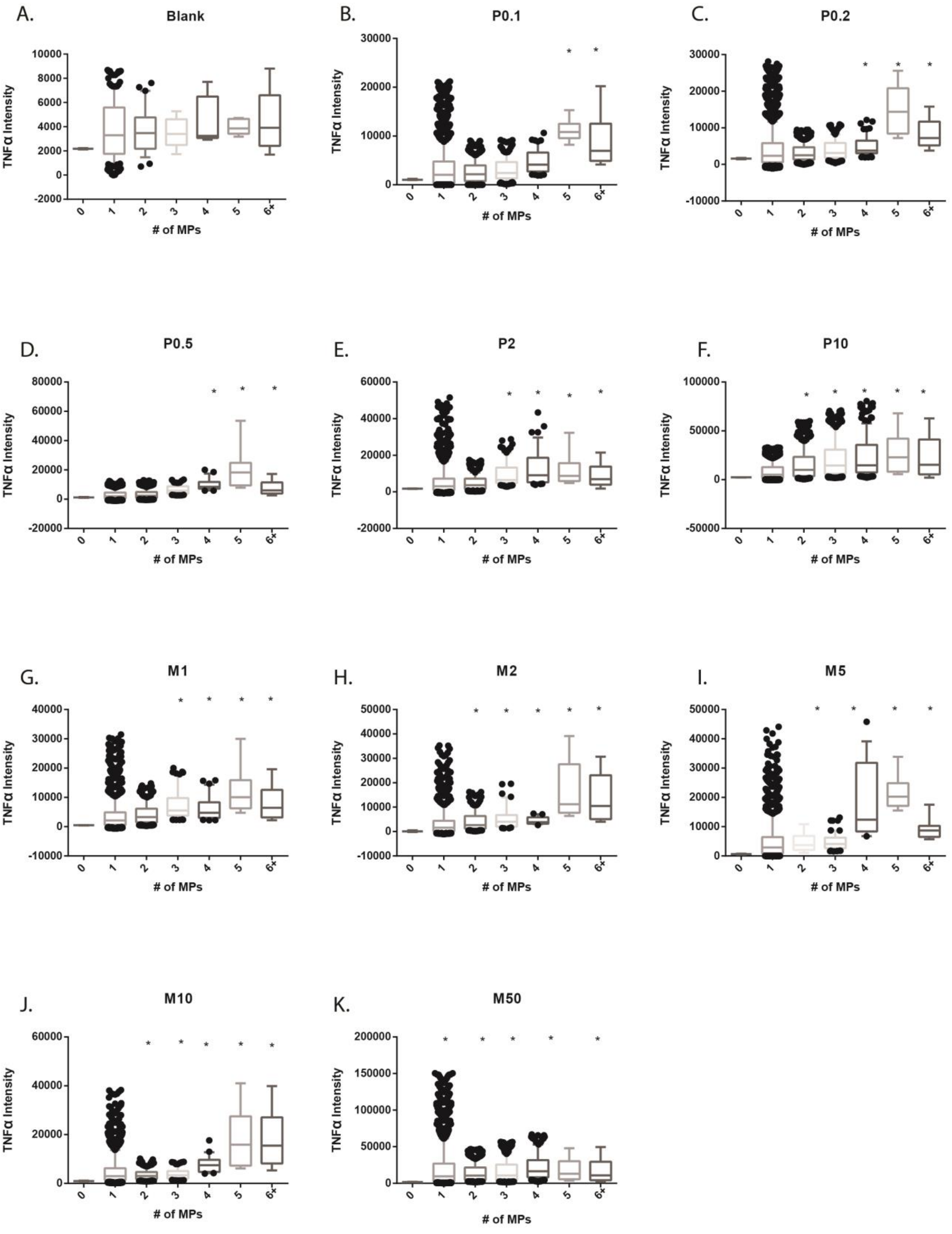

Figure S-8. THP-1s increase TNFα expression upon MP^{TLR} uptake. 10 million THP-1 were incubated with 2 million varying MPs overnight in 1 ug/mL brefeldin A. THP-1s were then washed,

fixed, permabilized, stained and analyzed with imagestream (>100,000 cells per run, done in triplicate then combined). TNFα expression was then compared to the number of particles cells uptake and compared to baseline TNFα (the average TNFα expression of unstimulated THP-1s). Error bars represent 90/10 % data range. Conditions with a significant ($p<0.05$) increase in TNFα when compared to unstimulated cells are marked with a star. The MPs used were (A) Blank MPs, (B) P0.1. (C) P0.2, (D) P0.5, (E) P2, (F)P10, (G) M1, (H) M2, (I) M5, (J) M10, (K) M50

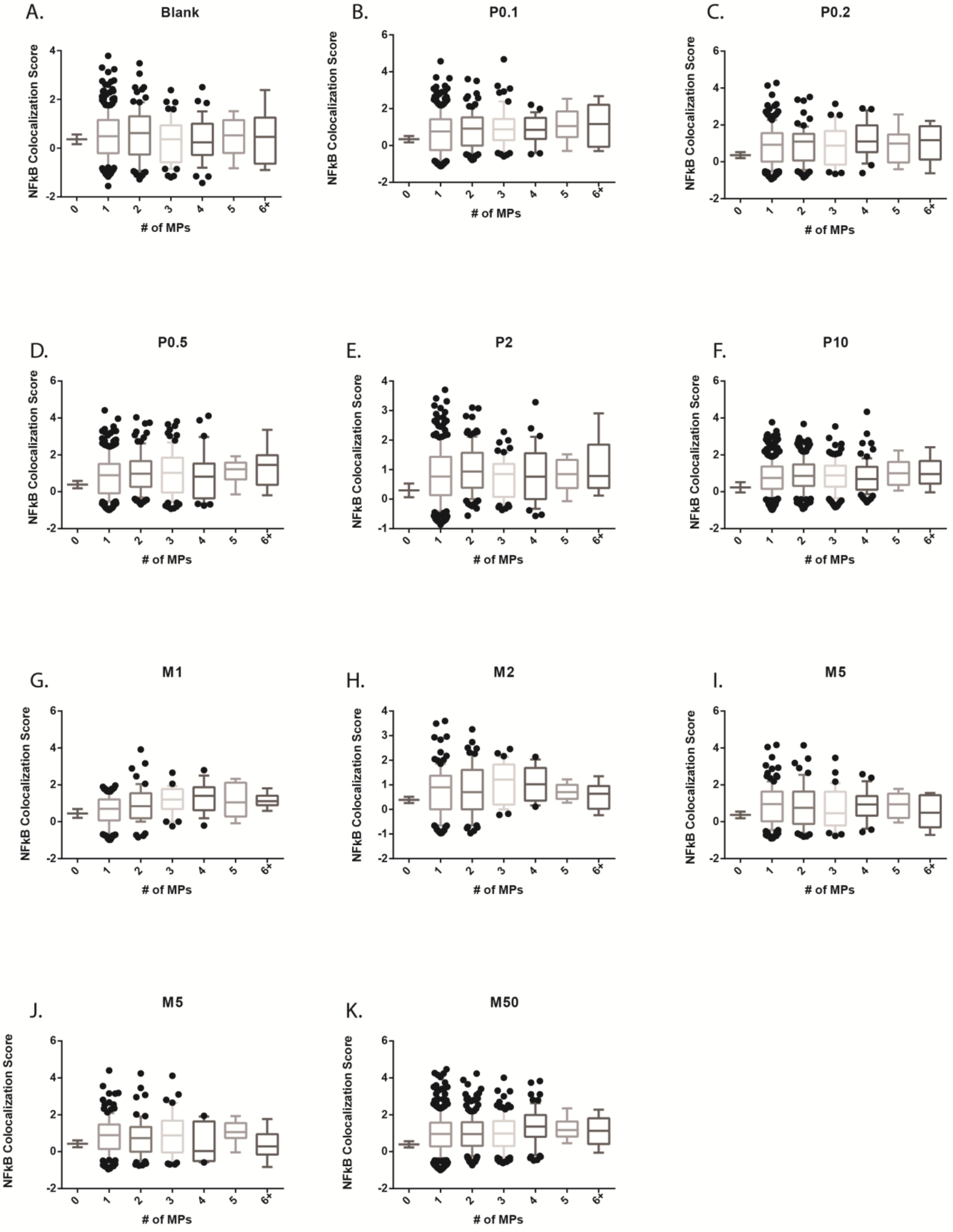

Figure S-9. BMDCs have increased NFkB nuclear colocalization when any cell uptakes a MP. 10 million BMDCs were incubated with 2 million varying MPs for 15 minutes. BMDCs were then

washed, fixed, permabilized, stained and analyzed with imagestream (>100,000 cells per run, done in triplicate then combined). NFkB colocalization was then compared to the number of particles cells uptake and compared to baseline NFkB colocalization (the average NFkB colocalization expression of unstimulated BMDCs). Error bars represent 90/10 % data range. Conditions with a significant ($p<0.05$) increase in NFkB colocalization when compared to unstimulated cells are marked with a star. The MPs used were (A) Blank MPs, (B) P0.1. (C) P0.2, (D) P0.5, (E) P2, (F)P10, (G) M1, (H) M2, (I) M5, (J) M10, (K) M50

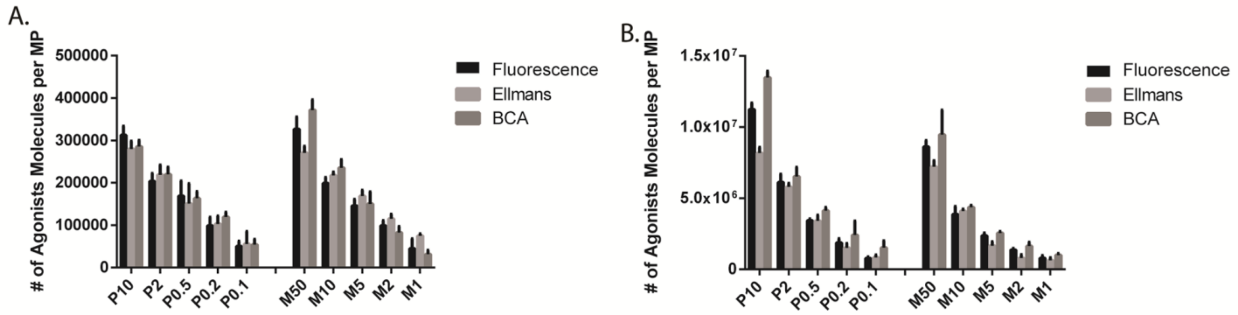

Figure S-10. Quantification of TLR conjugation for 0.25 and 5 μm diameter MPs. After conjugating MPLA or Pam2 to 0.25 or 5 μm diameter MPs and washing to remove unreacted TLR agonists, MPs were tested with the three analytical quantification methods, Fluorescence, Ellmans or BCA. All ratios of MPLA or Pam2 conjugated MPs were tested. (A) 0.25 μm MPs and (B) 5 μm MPs.

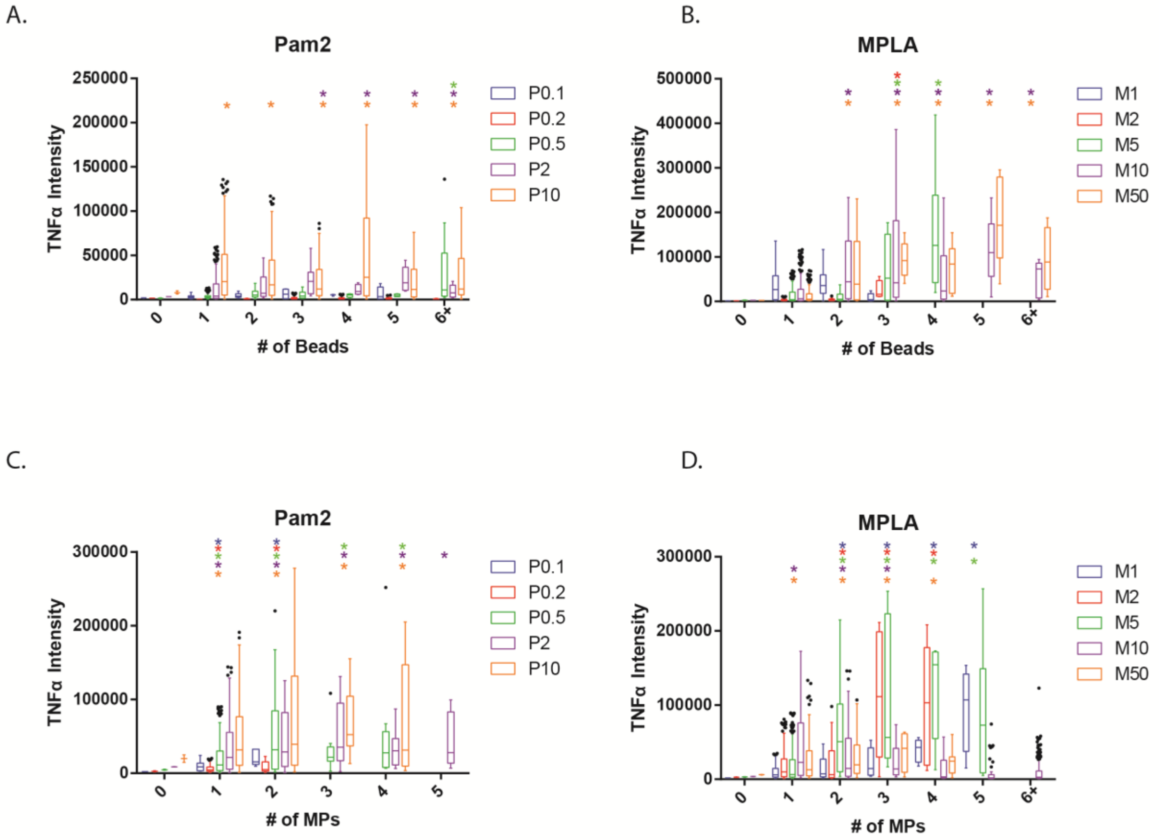

Figure S-11. BMDCs uptake 0.25 and 5 μm diameter MPs and trigger TNFα secretion. 10 million BMDCs were incubated with 2 million 0.25 μm diameter MPs or 5 μm diameter MPs overnight in 1 ug/mL brefeldin A. BMDCs were then washed, fixed, permabilized, stained and analyzed with imagestream (>100,000 cells per run, done in triplicate then combined). TNFα expression was then compared to the number of particles cells uptake and compared to baseline TNFα (the average TNFα expression of unstimulated BMDCs). Error bars represent 90/10 % data range. Conditions with a significant ($p<0.05$) increase in TNFα when compared to unstimulated cells are marked with a star with the matching color of the MPs. (A) Pam2 0.25 μm diameter MPs, (B) MPLA 5 μm diameter MPs, (C) Pam2 5 μm diameter MPs, (D) MPLA 5 μm diameter MPs.

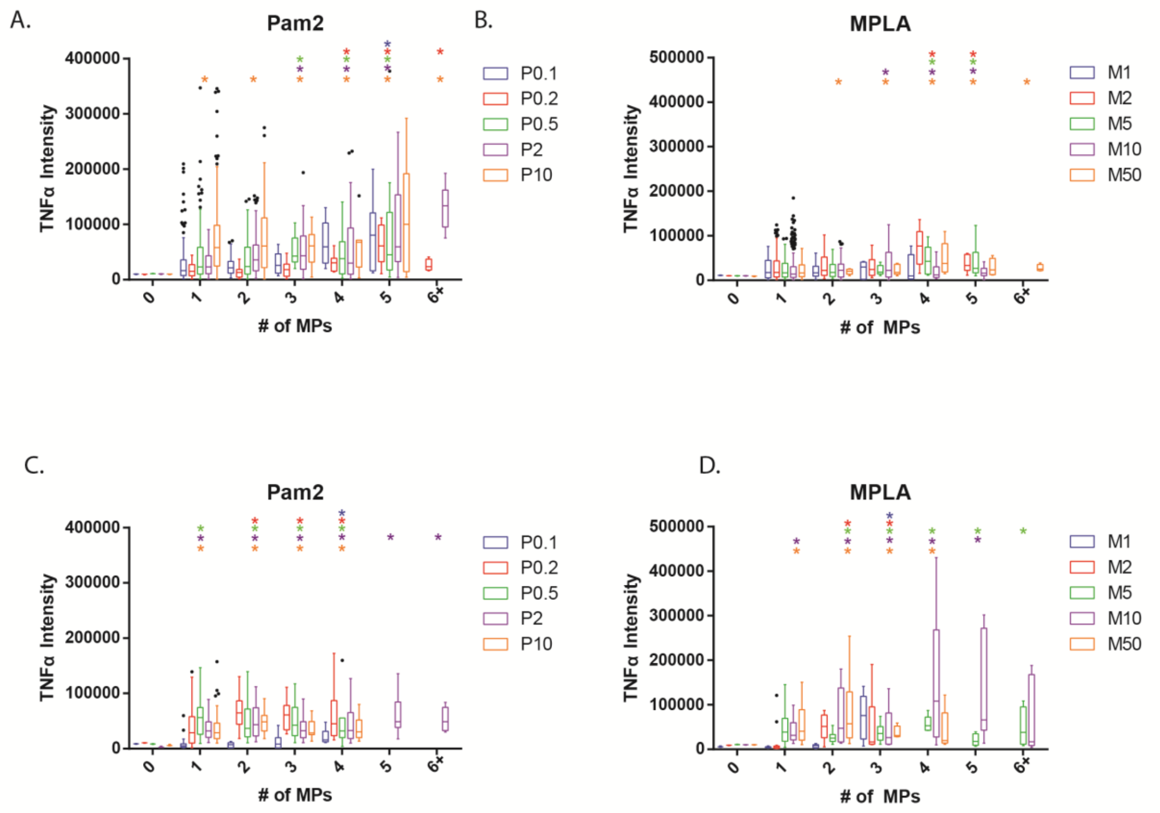

Figure S-12. RAW 264.7s uptake 0.25 and 5 µm diameter MPs and trigger TNFα secretion. 10 million RAWs were incubated with 2 million 0.25 µm diameter MPs or 5 µm diameter MPs overnight in 1 ug/mL brefeldin A. RAW s were then washed, fixed, permabilized, stained and analyzed with imagestream (>100,000 cells per run, done in triplicate then combined). TNFα expression was then compared to the number of particles cells uptake and compared to baseline TNFα (the average TNFα expression of unstimulated RAW s). Error bars represent 90/10 % data range. Conditions with a significant ($p<0.05$) increase in TNFα when compared to unstimulated cells are marked with a star with the matching color of the MPs. (A) Pam2 0.25 µm diameter MPs, (B) MPLA 5 µm diameter MPs, (C) Pam2 5 µm diameter MPs, (D) MPLA 5 µm diameter MPs.

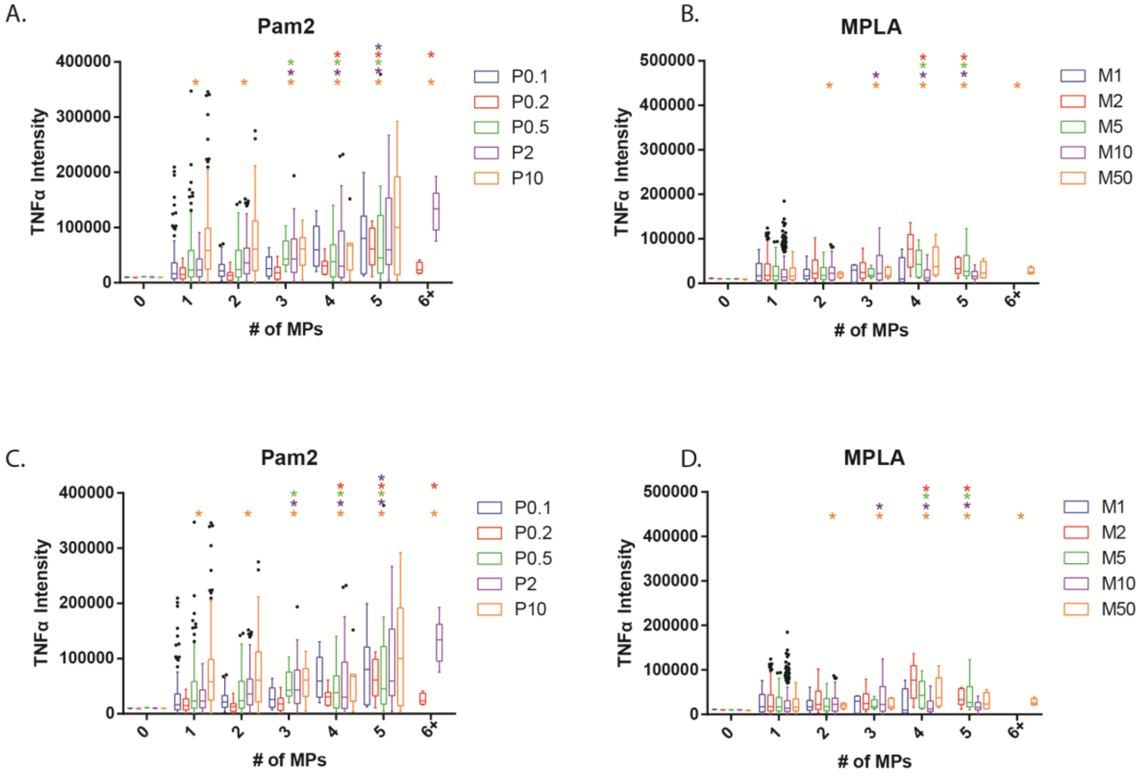

Figure S-13. THP-1s uptake 0.25 and 5 μm diameter MPs and trigger TNFα secretion. 10 million THP-1s were incubated with 2 million 0.25 μm diameter MPs or 5 μm diameter MPs overnight in 1 ug/mL brefeldin A. THP-1s were then washed, fixed, permabilized, stained and analyzed with imagestream (>100,000 cells per run, done in triplicate then combined). TNFα expression was then compared to the number of particles cells uptake and compared to baseline TNFα (the average TNFα expression of unstimulated THP-1s). Error bars represent 90/10 % data range. Conditions with a significant ($p<0.05$) increase in TNFα when compared to unstimulated cells are marked with a star matching the color of the MPs in the legend. (A) Pam2 0.25 μm diameter MPs, (B) MPLA 5 μm diameter MPs, (C) Pam2 5 μm diameter MPs, (D) MPLA 5 μm diameter MPs.

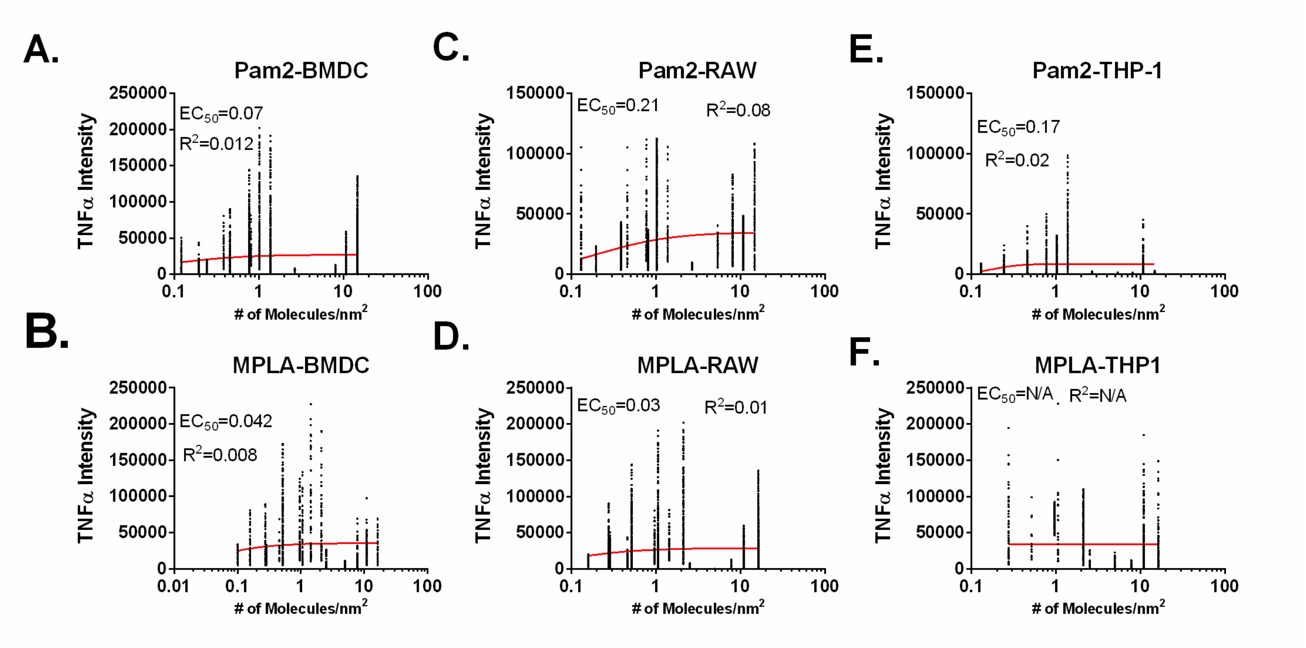

Figure S-14. TNFα expression is poorly correlated with TLR agonist density on cells that uptake MPs. By further analyzing the imagestream data, the TNFα expression for cells that uptake one MP was compared with the density of TLR agonist for each MP condition (calculated from the number of molecules on the MP surface and the surface area, estimated from the MP diameter). (A)BMDC cells stimulated with Pam2 conjugated MPs, (B) BMDCs stimulated with MPLA conjugated MPs, (C) RAWs stimulated with Pam2 conjugated MPs, (D) RAWs stimulated with MPLA conjugated MPs, (E) THP-1 simulated with Pam2 conjugated MPs, (F) THP-1 simulated with MPLA conjugated MPs.

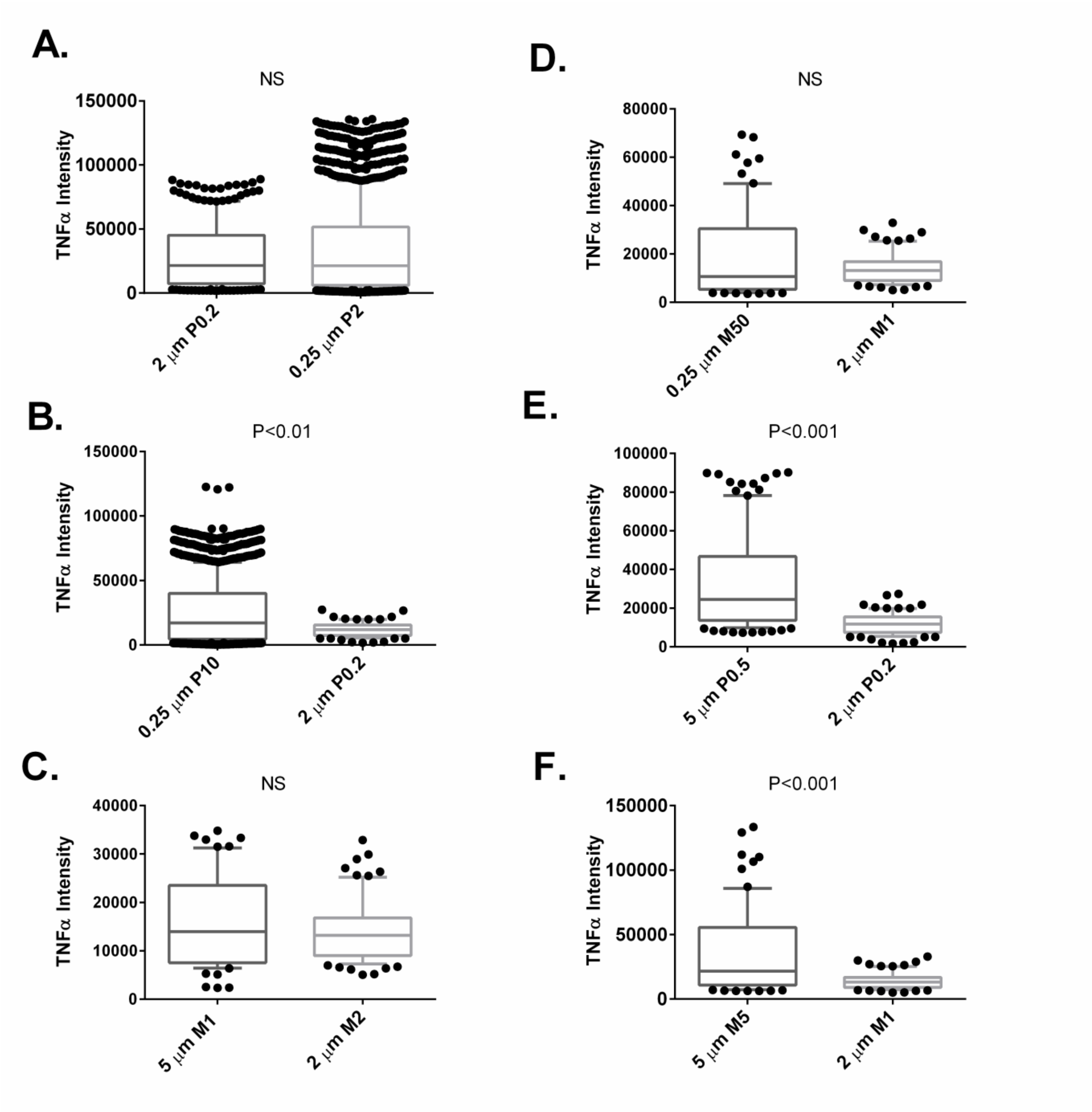

Figure S-15. Comparison between specific MP conditions demonstrate that total number of TLR agonists on MP surfaces is better correlated with TNFα intensity than TLR agonists density. We selected two MP formulated that either had similar total number of TLR agonists per MP (A, B, and C) or similar TLR agonist density (D, E, F) and compared the levels of TNFα secretion when BMDCs uptake a single MP. Error bars represent 90/10 % data range. Statistical difference between the two group is shown above graph, if $p>0.05$ then it is not significant (NS).

Table S-1. Total Agonist Number and Density for all MP formulations

**MP Diameter**	**0.25 µm**				**2 µm**				**5 µm**			
**MP Name**	**# of Agonist Molecules**	**SD**	**Agonist Density (mol/nm²)**	**SD**	**# of Agonist Molecules**	**SD**	**Agonist Density (mol/nm²)**	**SD**	**# of Agonist Molecules**	**SD**	**Agonist Density (mol/nm²)**	**SD**
**P10**	2.92E+05	1.74E+04	1.49	0.09	1.30E+06	2.61E+05	0.10	0.02	1.10E+07	2.66E+06	0.14	0.03
**P2**	2.14E+05	9.21E+03	1.09	0.05	1.02E+06	1.97E+05	0.08	0.02	6.14E+06	3.71E+05	0.08	0.00
**P0.5**	1.61E+05	9.26E+03	0.82	0.05	4.90E+05	2.71E+05	0.04	0.02	3.64E+06	4.06E+05	0.05	0.01
**P0.2**	1.07E+05	1.08E+04	0.54	0.05	2.48E+05	4.86E+04	0.02	0.00	1.93E+06	4.56E+05	0.02	0.01
**P0.1**	5.31E+04	2.92E+03	0.27	0.01	1.55E+05	1.54E+04	0.01	0.00	1.03E+06	4.18E+05	0.01	0.01
**M50**	3.23E+05	5.06E+04	1.65	0.26	2.68E+06	5.17E+05	0.21	0.04	8.42E+06	1.14E+06	0.11	0.01
**M10**	2.17E+05	1.83E+04	1.11	0.09	1.82E+06	8.19E+04	0.15	0.01	4.11E+06	2.48E+05	0.05	0.00
**M5**	1.55E+05	1.22E+04	0.79	0.06	1.22E+06	4.52E+05	0.10	0.04	2.19E+06	4.74E+05	0.03	0.01
**M2**	9.87E+04	1.64E+04	0.50	0.08	5.84E+05	3.29E+05	0.05	0.03	1.26E+06	4.27E+05	0.02	0.01
**M1**	5.02E+04	2.23E+04	0.26	0.11	3.64E+05	2.35E+05	0.03	0.02	8.02E+05	1.87E+05	0.01	0.00
